# The effect of Ramadan on forensic case admissions to the emergency department: A five-year retrospective analysis in Turkiye

**DOI:** 10.12669/pjms.41.11.12034

**Published:** 2025-11

**Authors:** Mustafa Alpaslan, Necmi Baykan, Omer Salt, Funda Ipekten

**Affiliations:** 1 Mustafa Alpaslan Specialist Doctor, Department of Emergency Medicine, Nevsehir Ministry and Health Center, 50100, Nevsehir, Turkiye; 2 Necmi Baykan Associate Professor, Department of Emergency Medicine, Health Sciences University, Kayseri Health Practice and Research Center, 38080, Kayseri, Turkiye; 3 Omer Salt Associate Professor, Department of Emergency Medicine, Health Sciences University, Kayseri Health Practice and Research Center, 38080, Kayseri, Turkiye; 4 Funda Ipekten Research Assistant, Department of Biostatistics and Medical Informatics, Adıyaman University Faculty of Medicine, 02040, Adıyaman, Turkiye

**Keywords:** Emergency Department, Forensic Case, Ramadan, Trauma, Traffic Accident, Occupational Injury, Suicid, Injury

## Abstract

**Objective:**

The aim of this study was to compare the patients who were evaluated as forensic cases in the emergency department during Ramadan according to the month before and after Ramadan and to examine the effect of this process on forensic case management.

**Methodology:**

This study was conducted retrospectively using patient data from the emergency department of Nevsehir State Hospital in Turkiye. Within the scope of the study, patients admitted to the emergency department between 2020 to 2024 who were recorded as forensic cases were evaluated. The demographic data of the patients, time of admission, reason for admission, discharge status in the emergency department and diagnoses of inpatients were analyzed.

**Results:**

In the study, 4020 forensic cases were evaluated. 69.4% of the patients were male. The mean age was 32.57±17.52 years. There were more admission (38.8%) after Ramadan. The most common reason for admission was traffic accidents (47.5%). There was no significant difference in the number of patient admissions between the defined periods (p=0.078). The number of patient admissions was significantly higher between the hours of Iftar to Imsak (p<0.001).

**Conclusion:**

Comparison between before and after Ramadan, the number of patient admissions evaluated as forensic cases increased significantly after Ramadan. There was no significant difference between the hours of admission. It was observed that there was a significant increase in traffic accident-related admissions close to Iftar time.

## INTRODUCTION

The month of Ramadan corresponds to the ninth month of the Islamic calendar, during which Muslims observe mandatory fasting throughout the month. The fasting period lasts approximately 12 to 16 hours, though this may vary between countries depending on geographical location and seasonal factors.^1^,^2^ According to the Gregorian calendar, Ramadan begins 11 days earlier each year than the previous year because the lunar year is 11 days shorter than the solar year (354 days versus 365 days).^3^

The act of fasting can cause changes in people’s daily rhythms and physiological functioning.^4^ Due to the many restrictions imposed during the day as required by the rules of worship, excessive food consumption at Iftar time, and changes in sleep patterns, people who fast may develop anxiety disorders and excessive irritability.^4^ While this situation may increase the tendency toward violence in people, on the other hand, the spiritual effect of Ramadan fasting may keep people away from violence.^4^ There may be an increase in traffic accidents during Ramadan. Due to sleep deprivation and reduced food and drink intake, drivers may experience decreased attention span and slowed physical and cognitive abilities, increasing the risk of collision.^5^,^6^ However, a study found that falls and violence-related traumas did not occur more frequently during Ramadan than in other months.^7^

In Turkiye, a forensic case is defined as any event resulting from external factors that causes harm to a person’s physical and mental health or death. Events such as traffic accidents, assault and battery, firearm injuries, stab wounds, and work accidents are considered forensic cases.^8^

Reviewing the literature, we observed that studies related to the month of Ramadan were more frequently conducted on internal pathologies and traffic accidents. In this study, we aimed to present new and different data to the literature by comparing the profile of patients evaluated as forensic cases in the emergency department during Ramadan comparing the months before and after Ramadan, thereby investigating the effect of this process on forensic case presentations.

## METHODOLOGY

**Fig.1:**
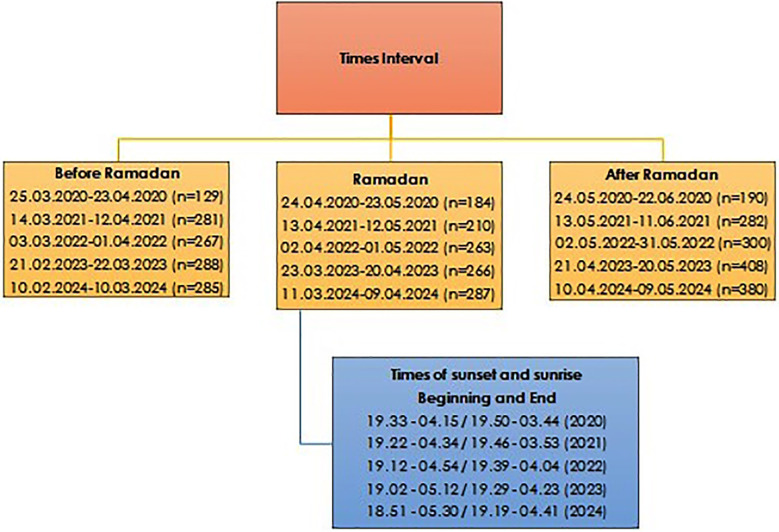
Time periods and patient numbers for the month of Ramadan, before and after Ramadan, from which the working data was obtained by year.

This study was conducted at the emergency department of Nevsehir State Hospital in Nevsehir, Turkiye. Within the scope of the study, patients who were recorded as forensic cases among those who visited the emergency department during the periods given in Fig.1 between 2020 to 2024 (a five-years period) were evaluated. Patient data included in the study were selected from the dates given in Fig.1. The dates given in Fig.1 indicate 30-day periods before and after the days of Ramadan for each year. The time intervals for sunrise (imsak) and sunset (Iftar) at the beginning and end of Ramadan are provided as additional data below the data indicating the Ramadan months in Fig.1. Data related to time zones and fasting calendar were obtained from the website of the Presidency of Religious Affairs of the Republic of Turkiye.^9^

Patient data were obtained through the hospital electronic data system. The patients’ age, gender, time of admission to the emergency department, reason for acceptance, outcome in the emergency department, and diagnoses of inpatients were analyzed. The times of admission to the emergency department were evaluated in three groups according to the date ranges indicated in Fig.1: Ramadan month, before Ramadan, and after Ramadan, based on the year, hour, and time interval. The time intervals were evaluated in two groups according to the time intervals indicated in Fig.1: from Iftar to imsak and from imsak to Iftar. During this process, the group to which the patient would be included was determined according to the fasting calendar times on the day of admission.

### Inclusion and exclusion criteria:

All patients evaluated as forensic cases during the specified time periods at the center where the study was conducted were included in the study. Since the center where the study was conducted operated as a separate pediatric internal emergency department, only traumatic forensic cases in the pediatric age group were evaluated. Patients diagnosed with routine assault and battery examination requested by law enforcement were not included in the study. Only the mortality of patients followed in the emergency department was evaluated, and patients transferred to external centers could not be followed up. Since the study was retrospective, detailed data on whether the patients included in the evaluation were fasting could not be obtained.

### Ethics committee approval:

Hacıbektas Veli University Non-Interventional Clinical Research Ethics Committee approval was obtained with decision number 2024/09; Dated: July 25, 2024.

### Statistical Analysis:

Statistical Package for Social Sciences for Windows 21.0 (SPSS 21.0) program was used to analyze the data. Descriptive statistics (frequency, percentage distribution) and Chi-square and Fischer Exact test were used for the comparison of categorical variables between two groups. Student T test, One Way Anova and/or Mann Whitney U test were used to compare independent groups. Results were presented as mean ± SD or frequency (percentage) and p<0.001 was considered statistically significant at 95 percent confidence interval.

## RESULTS

This study evaluated 4,020 forensic cases of which 69.4% of patients were male. The mean age was 32.57±17.52, with the most common age group being 21-40 years old, comprising 1,882 patients (46.8%). Looking at the distribution of cases by year, the lowest number of cases occurred in 2020 (12.5%), while the highest number of cases were admitted in 2023 (23.9%). When patient cases were evaluated in general before and after Ramadan, it was seen that there were more cases after Ramadan, with a rate of 38.8%. The most common reason for admission was injuries resulting from traffic accidents (47.5%). It was observed that 9.3% of patients were hospitalized for treatment. The most common reasons for hospitalization were lower extremity fractures (27.2%), drug intoxication (22.1%), and intracranial hemorrhage (11.9%). It was also observed that 90.1% of the patients were discharged from the emergency department. The general distribution of data for the period before and after Ramadan is presented in Table-I. The distribution of patient visit reasons by year and before and after Ramadan is presented in Table-II.

**Table-I:** Distribution of data by month of Ramadan, before and after.

Datas	Period			Total (n/%)	Statistical Test	P Value*
Gender	Before (n/%)	Ramadan (n/%)	After (n/%)			
Male	813 (29.1)	859 (30.8)	1118 (40.1)	2790 (69.4)	x^2^=16.408	<0.001
Female	437 (35.5)	351 (28.5)	442 (35.9)	1230 (30.6)		
Average age	33.19±16.92	32.88±17.92	31.83±17.66	32.57±17.52	F=2.362	0.094
***Age Range***						
0-20	270 (27.2)	307 (30.9)	415 (41.8)	992 (24.7)	x^2^=11.586	0.171
21-40	619 (32.9)	549 (29.2)	714 (37.9)	1882 (46.8)		
41-60	260 (31.7)	253 (30.9)	306 (37.4)	819 (20.4)		
61-80	85 (30.1)	88 (31.2)	109 (38.7)	282 (7.0)		
81-100	16 (35.6)	13 (28.9)	16 (35.6)	45 (1.1)		
***Admitted Year***						
2020	129 (25.6)	184 (36.6)	190 (37.8)	503 (12.5)	x^2^=30.900	<0.001
2021	281 (36.4)	210 (27.2)	282 (36.5)	773 (19.2)		
2022	267 (32.2)	263 (31.7)	300 (36.1)	830 (20.6)		
2023	288 (29.9)	266 (27.7)	408 (42.4)	962 (23.9)		
2024	285 (29.9)	287 (30.1)	380 (39.9)	952 (23.7)		
***Reason for admitted***						
Traffic accident	545 (28.5)	569 (29.8)	795 (41.6)	1909 (47.5)	x^2^=137.693	<0.001
Occupational injury	211 (30.4)	265 (38.2)	218 (31.4)	694 (17.3)		
Fall from height	105 (26)	112 (27.7)	187 (46.3)	404 (10.0)		
Suicid	109 (35.4)	76 (24.7)	123 (39.9)	308 (7.7)		
Penetrating and sharp instrument injury	62 (32)	58 (29.9)	74 (38.1)	194 (4.8)		
Carbon monoxide poisoning	106 (55.8)	54 (28.4)	30 (15.8)	190 (4.7)		
Assult	21 (42)	10 (20)	19 (38)	50 (1.2)		
Burn	10 (25)	11 (27.5)	19 (47.5)	40 (1.0)		
Firearm injury	15 (37.5)	10 (25)	15 (37.5)	40 (1.0)		
Food poisoning	12 (31.6)	11 (28.9)	15 (39.5)	38 (0.9)		
Animal bite	11 (29.7)	7 (18.9)	19 (51.4)	37 (0.9)		
Amputation	8 (22.2)	12 (33.3)	16 (44.4)	36 (0.9)		
Alcohol use	20 (55.6)	3 (8.3)	13 (36.1)	36 (0.9)		
Electric shock	7 (33.3)	6 (28.6)	8 (38.1)	21 (0.5)		
Behavior disorder and aggression	4 (40)	1 (10)	5 (50)	10 (0.2)		
Uncaused arrest	3 (33.3)	4 (44.4)	2 (22.2)	9 (0.2)		
Drawning in water	1 (25)	1 (25)	2 (50)	4 (0.1)		
***Diagnoses in inpatients***						
Lower extremity fracture	22 (19.6)	43 (38.4)	47 (42)	112 (27.2)	x^2^=29.009	0.413
Drug intoxication	32 (35.2)	23 (25.3)	36 (39.6)	91 (22.1)		
Intracranial hemorrhage	12 (24.5)	17 (34.7)	20 (40.8)	49 (11.9)		
Intra-abdominal organ injury	12 (31.6)	10 (26.3)	16 (42.1)	38 (9.24)		
Hemopneumothorax	10 (27)	14 (37.8)	13 (35.1)	37 (9.00)		
Vertebral fracture	8 (36.4)	7 (31.8)	7 (31.8)	22 (3.89)		
Upper extremity injury	5 (31.3)	5 (31.3)	6 (37.5)	16 (3.89)		
Threat of miscarriage in pregnancy	3 (30)	2 (20)	5 (50)	10 (2.43)		
Tendon injury	6 (66.7)	1 (11.1)	2 (22.2)	9 (2.18)		
Amputation	3 (37.5)	2 (25)	3 (37.5)	8 (1.94)		
Hypoxy	3 (50)	2 (33.3)	1 (16.7)	6 (1.45)		
Peripheral vascular injury	2 (40)	1 (20)	2 (40)	5 (1.21)		
Methanol poisoning	3 (75)	0 (0)	1 (25)	4 (0.97)		
Corneal perforation	2 (100)	0 (0)	0 (0)	2 (0.48)		
Burning	0 (0)	1 (50)	1 (50)	2 (0.04)
***Hospitalization***						
Discharced	1137 (31.4)	1084 (29.9)	1401 (38.7)	3622 (90.1)	x^2^=6.669	0.573
Service	57 (26.1)	74 (33.9)	87 (39.9)	218 (5.4)		
Intensive care unit	48 (30.2)	47 (29.6)	64 (40.3)	159 (4.0)		
Referral to another center	8 (40)	4 (20)	8 (40)	20 (0.5)		
Exitus	0 (0)	1 (100)	0 (0)	1 (0.0)		
Total	1250 (31.1)	1210 (30.1)	1560 (38.2)	4020 (100)	

* The chi-square and Fisher’s exact tests were used to compare categorical variables between the groups. The One-Way ANOVA test was used to analyze the comparison of independent groups.

**Table-II:** Distribution of the reasons for patient admitted during, before and after Ramadan according to years.

Year	Period	Traffic Accident	Occupational Injury	Fall From a Height	Suicid	Penetrating and Sharp Instrument Injury	Carbon Monoxide Poisoning	Assault	Burn	Firearm Injury	Food Poisoning	Amputation	Animal Bite	Alcohol use	Electrıc Shock	Behavior Disorder and Aggression	Uuncaused arrest	Drowning in water	Total	Statistical test	P Value*
2020	Before	29(22.5)	24(18.6)	21(16.3)	15(11.6)	14(10.9)	11(8.5)	5(3.9)	1(0.8)	1(0.8)	0(0)	2(1.6)	1(0.8)	5(3.9)	0(0)	0(0)	0(0)	0(0)	129(100)	x^2^=58.974	<0.001
	Ramadan	61(33.2)	40(21.7)	22(12.0)	12(6.5)	19(10.3)	11(6.0)	0(0)	2(1.1)	2(1.1)	4(2.2)	7(3.8)	2(1.1)	0(0)	2(1.1)	0(0)	0(0)	0(0)	184(100)		
	After	79(41.6)	27(14.2)	35(18.4)	14 (7.4)	14 (7.4)	7 (3.7)	0(0)	1(0.5)	2(1.1)	5(2.6)	4(2.1)	2(1.1)	0(0)	0(0)	0(0)	0(0)	0(0)	190(100)		
2021	Before	108(38.4)	34(12.1)	23(8.2)	22(7.8)	11(3.9)	54(19.2)	4(1.4)	2(0.7)	6(2.1)	3(1.1)	0(0)	3(1.1)	8(2.8)	3(1.1)	0(0)	0(0)	0(0)	281(100)	x^2^=138.442	<0.001
	Ramadan	87(41.4)	54(25.7)	26(12.4)	15(7.1)	11(5.2)	3(1.4)	6(2.9)	1(0.5)	1(0.5)	3(1.4)	0(0)	3(1.4)	0(0)	0(0)	0(0)	0(0)	0(0)	210(100)		
	After	122(43.3)	33(11.7)	46(16.3)	35(12.4)	12(4.3)	0(0)	6(2.1)	5(1.8)	2(0.7)	5(1.8)	0(0)	10(3.5)	3(1.1)	3(1.1)	0(0)	0(0)	0(0)	282(100)		
2022	Before	126(47.2)	58(21.7)	15(5.6)	18(6.7)	7(2.6)	28(10.5)	1(0.4)	0(0)	4(1.5)	0(0)	0(0)	3(1.1)	2(0.7)	1(0.4)	4(1.5)	0(0)	0(0)	267(100)	x^2^=94.198	<0.001
	Ramadan	118(44.9)	59(22.4)	28(10.6)	19(7.2)	6(2.3)	18(6.8)	1(0.4)	3(1.1)	3(1.1)	1(0.4)	0(0)	2(0.8)	0(0)	3(1.1)	1(0.4)	1(0.4)	0(0)	263(100)		
	After	170(56.7)	31(10.3)	39(13)	31(10.3)	18(6.0)	1(0.3)	5(1.7)	1(0.3)	5(1.7)	5(1.7)	0(0)	0(0)	2(0.7)	0(0)	3(1)	0(0)	0(0)	300(100)		
2023	Before	153(53.1)	35(12.2)	22(7.6)	23(8)	17(5.9)	6(2.1)	11(3.8)	5(1.7)	0(0)	0(0)	6(2.1)	4(1.4)	3(1)	1(0.3)	0(0)	1(0.3)	1(0.3)	288(100)	x^2^=47.195	0.019
	Ramadan	145(54.5)	54(20.3)	14(5.3)	15(5.6)	10(3.8)	11(4.1)	2(0.8)	4(1.5)	3(1.1)	0(0)	4(1.5)	0(0)	1(0.4)	1(0.4)	0(0)	(1(0.4)	1(0.4)	266(100)		
	After	203(49.8)	77(18.9)	39(9.6)	22(5.4)	18(4.4)	12(2.9)	0(0)	8(2.0)	3(0.7)	0(0)	9(2.2)	5(1.2)	4(1.0)	3(0.7)	2(0.5)	1(0.2)	2(0.5)	408(100)		
2024	Before	129(45.2)	60(21.1)	24(8.4)	31(10.9)	13(4.6)	7(2.5)	0(0)	2(0.7)	4(1.4)	9(3.2)	0(0)	0(0)	2(0.7)	2(0.7)	0(0)	2(0.7)	0(0)	285(100)	x^2^=48.929	0.004
	Ramadan	158(55.1)	58(20.2)	22(7.7)	15(5.2)	12(4.2)	11(3.8)	1(0.3)	1(0.3)	1(0.3)	3(1)	1(0.3)	0(0)	2(0.7)	0(0)	0(0)	2(0.7)	0(0)	287(100)		
	After	221(58.2)	50(13.2)	28(7.4)	36(9.5)	12(3.2)	10(2.6)	4(1.1)	4(1.1)	3(0.8)	0(0)	3(0.8)	2(0.5)	4(1.1)	2(0.5)	0(0)	1(0.3)	0(0)	380(100)		

* The chi-square and Fisher’s exact tests were used to compare categorical variables between the groups.

**Fig.2:**
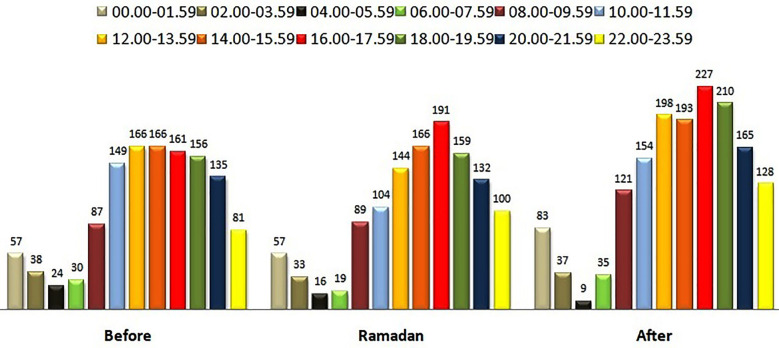
Distribution of patient admissions by time intervals before and after Ramadan.

The number of patient visits was separated by time slots and presented in Fig.2 according to the period before and after Ramadan. The comparison showed no significant difference between the periods (*P*=0.078). In general, patient visits were most frequent between 12:00 and 17:59 (Fig.2). During Ramadan, the highest patient visit (15.7%) occurred between 16:00 and 17:59. Patient visits were analyzed by year, comparing the period between imsak to Iftar times and the period between Iftar to Imsak times. Accordingly, in all years, the number of patient visits was significantly higher between imsak to Iftar times (*P*<0.001). The admissions due to traffic accident, which were the most common reasons were analyzed according to time slots, and a significant difference was observed between visit times (*P*<0.001). Overall, the most frequent visits occurred between 18:00 and 18:59, accounting for 10.5% of visits, while the least frequent visits occurred between 05:00 and 05:59, accounting for 0% of visits. During Ramadan, the highest number of traffic accident cases (10.1%) occurred between 18:00 and 18:59, while the lowest number of accidents (0%) occurred between 05:00 and 05:59. There was no significant difference in the time of admission between patients admitted due to traffic accidents before Ramadan and during Ramadan (*P*=0.281) or between Ramadan and the following month (*P*=0.232).

Patient visits due to traffic accidents were most frequent between 16:00-16:59 (9.3%) before Ramadan, between 18:00-18:59 (10.1%) during Ramadan, and between 18:00-18:59 (9.4%) after Ramadan. A significant difference was observed in the comparison of the reasons for patient visits to the emergency department according to time intervals (*P*<0.001). According to the analysis of the general data, the most frequent reason for visits to the emergency department was traffic accidents, which occurred in 54% of cases between 16:00 and 17:59, 31.2% of work accident cases between 10:00 a.m. and 11:59 a.m., and 12.6% of falls from height cases between 6:00 p.m. and 7:59 p.m. (*P*<0.001). Looking at the cases during Ramadan, it was observed that there were significantly more cases of traffic accidents (18.2%) between 6:00 p.m. and 7:59 p.m., work accidents (20.9%) between 12:00 p.m. and 1:59 p.m., and falls from heights (21%) between 12:00 p.m. and 1:59 p.m. (*P*<0.001). When analyzing the diagnoses given to patients according to the time of application, no significant differences were found in the analysis of the general data (*P*=0.045) or in the analysis of the data during Ramadan (*P*=0.999).

According to the comparative analyses in our study, the legal case was significantly higher among males (*P*<0.001). The female involved in accidents during Ramadan (%28.5) was significantly lower than in other months (*P*<0.001). While suicide attempts (%59) and carbon monoxide poisoning rates (65.2 %) were higher among women, men were more common in other cases. When examining the number of legal cases by age group, a significant difference was observed (*P*<0.001). Accordingly, the majority of cases, primarily traffic accident cases (43.7%), were among those aged 21-40. Amputation (47.2%) and drowning (75%) cases were more common in the 0-20 age group, while arrests of unknown cause were more common in the 41-60 age group (55.6%).

According to the study data, 74.1% of lower extremity fractures, 51% of intracranial hemorrhage cases, 70.3% of cases with hemopneumothorax, and 39.5% of intra-abdominal organ injuries related to traffic accident. When comparing the reasons for admission and the outcomes of patients discharged from the emergency department, significant difference were observed (*P*<0.001). As a result, the highest discharge rate was observed in traffic accident cases (47.8%), while the highest rates of admission to the ward (47.7%) and intensive care (44%) were also associated with traffic accident cases.

## DISCUSSION

Forensic cases are extraordinary circumstances that arise from external factors and cause physical and psychological disorders in a person’s health. Emergency departments are among the most frequent referral sites for events considered forensic cases in daily life.^10^ Ramadan is considered one of the most sacred and important religious events for Muslim communities. Strict rituals and commandments are observed every year during this period.^11^ The changes in eating and drinking habits and sleep patterns that occur during this period can lead to conditions such as absent-mindedness, inattention, and a tendency towards violence.^4^

We observed that studies in the literature regarding the effects of Ramadan on health factors include studies on pregnancy, demographic data in emergency department visits, and analyses of internal diseases and traffic accidents.^1^-^7^,^11^-^13^ The aim of this study was to examine whether there are differences in the number of patients admitted to the emergency department as forensic cases during Ramadan between the months before and after Ramadan and to examine the impact of Ramadan on emergency department forensic case visits.

The study found that the number of patients considered forensic cases was lower than before and after Ramadan. A similar study noted a general decrease in emergency department visits during Ramadan, and a significant decrease in the number of forensic cases during Ramadan.^4^ Some studies in the literature indicate a decrease in emergency department visits during Ramadan.^4^,^11^,^14^ When we examined the distribution of patients by year in the study, we observed an increase in the number of cases after Ramadan compared to the previous month. We believe this is due to people restricting themselves for about a month, focusing more on religious activities, and subsequently re-entering social life as if they had regained some freedom.

When we look at the study’s demographic data, the proportion of male cases was 69.4% higher. The most frequent visits were between the ages of 21 and 40. Studies on emergency department visits during Ramadan have shown a higher proportion of male patient.^1^,^2^,^14^,^15^ Because this study evaluated isolated forensic cases, we believe the proportion of male patients and young-middle-aged patients is higher than in similar studies. Indeed, studies on forensic cases presenting to the emergency department in the literature have also yielded results indicating a significantly higher proportion of male patients and a higher proportion of middle-aged patients presenting.^8^,^10^,^16^

The number of emergency department visits during Ramadan may vary compared to other months. A study conducted in Turkiye reported that the number of patient visits during Ramadan is lower in hours around Iftar compared to other months, with a significant increase after Iftar time.^4^ Furthermore, a study conducted in the same month reported a higher number of visits during the pre-dawn meal.^4^ A study conducted in Saudi Arabia found an increase in visits during the night shift during Ramadan compared to other months.^17^ This study found that emergency department visits for forensic cases during Ramadan were similar to the previous and subsequent months.

A review of the literature revealed that traffic accidents, workplace accidents, and suicide attempts are the most common forensic cases in emergency department visits.^4^,^8^,^16^ This study also found that traffic accident-related visits were the most common forensic cases. Şimsek et al. reported a decrease in the number of traffic accidents and workplace accidents during Ramadan compared to the previous and subsequent months, but there was no statistical difference.^4^ In a recent 2025 study, Zohrevandi et al. reported a significant increase in the incidence of traffic accidents during Ramadan compared to other months.^18^

A 2024 systematic review of the literature on the effects of fasting on trauma cases found that nine studies addressed traffic accidents, six of which reported a higher rate of traffic accidents during Ramadan.^7^ Furthermore, three studies reported higher rates of traffic accidents at sunset during Ramadan.^7^ Alsofayan et al. analyzed nationwide data from patients presenting to emergency departments after traffic accidents in Saudi Arabia and reported that 8.8% of accidents occurred during Ramadan.^5^ The same study reported that the rate of traffic accidents occurring at sunset was higher during Ramadan compared to other months, and the mortality rate associated with traffic accidents was significantly higher during Ramadan.^5^

This study also found an increase in the number of traffic accident-related visits compared to the previous month, but no significant difference was found. When considering admission times, the highest rate of traffic accident admissions during Ramadan was between 6:00 p.m. and 6:59 p.m., representing 10.1% of admissions. Similar to other studies, accident were higher at sunset, but no significant difference was found between the pre-Ramadan and post-Ramadan admission times. Considering the results of our study and literature data, an increase in the number of traffic accidents can be expected around sunset, the end of the fast. This is the time when patients go the longest without eating. Low blood sugar, a tendency to rush to break their fast, and heavy traffic during rush hour may explain the increase in traffic accidents at sunset.^5^

In our study, we found that occupational injuries ranked second among legal claims. There were more occupational injury claims during Ramadan than in the preceding and following months. Ricc`o et al. analyzed data on occupational accidents in an Italian province over a 13-years period and reported that an overall rate of 8.9% of occupational accidents occurred during Ramadan.^19^ This study found that the occupational accident rate during Ramadan was higher among people from the Eastern Mediterranean than in other months, with more accidents occurring between 1:00 p.m. and 6:00 p.m..^19^ A study conducted in Spain reported a 4% decrease in occupational injury rates during Ramadan among Muslim workers compared to first-generation non-Muslim immigrants, based on daily observations from 2003 to 2016.^20^ Studies show that occupational accidents increase or decrease during Ramadan.^19^,^20^

One study suggested that the risk of trauma may increase in the final phase of fasting due to decreased blood sugar, decreased attention span, and weakened reflexes, but it was emphasized that further studies are needed to confirm this claim.^7^ Our study found a decrease in suicide attempts, assaults, gunshot wounds, stab wounds, and injuries related to sharp objects, as well as alcohol consumption, during Ramadan compared to the previous and subsequent months. A study conducted in Turkiye analyzed monthly national suicide statistics in Turkiye between 2003 and 2022 to determine whether there was a statistically significant difference in suicide rates between the months during and without Ramadan. Consequently, suicide rates were found to be significantly lower in the total population and among the male population during Ramadan.^21^

In this study, Kartal et al. emphasized that religious sentiment and social environments during Ramadan may protect Muslims from suicide.^21^ A study on the effects of fasting during Ramadan on mood observed a slight increase in anger during fasting showed that anxiety and depression gradually decreased.^22^ The same study noted emotional improvement as Ramadan progressed, particularly after the fast ended.^22^ A study by Şimsek et al. similarly observed a decrease in alcohol-related visits.^4^ A study conducted in Adana, Turkiye, observed a significant decrease in illicit substance use and alcohol use during Ramadan compared to other months.^23^ Mohseni et al. reported a significant decrease in emergency department visits related to violence, such as gunshot and knife injuries, during Ramadan. 24 Our study found similar results to the literature in this respect.

The number of studies on the general evaluation of forensic case visits to the emergency department during Ramadan is quite limited. We believe this study will contribute to the literature. We would like to emphasize that more comprehensive research on this topic would be beneficial. Because we think that the fasting period of Muslims living in different countries, the Iftar and imsak times, and the seasons in which Ramadan is observed will vary.

## CONCLUSIONS

The study found a significant increase in the number of patient visits evaluated as forensic cases during the pre-Ramadan and post-Ramadan periods. No significant difference was observed between visit times. A significant increase in visits related to traffic accidents was observed around Iftar time. Public awareness by responsible organizations in Muslim countries about the increase in traffic accidents and related injuries during Ramadan could be effective in reducing accidents. Traffic safety organizations should educate the public about increased driving behavior during this month, and traffic and speed controls should be monitored more comprehensively, especially around Iftar time. The number related to workplace accidents increased during Ramadan. In this regard, we would like to emphasize the need for adjustments to working hours during Ramadan and the need for occupational health and safety professionals to take measures to reduce accidents.

### Author contributions:

**MA:** Conception, design and is responsible for the accuracy and integrity of this work.

**MA and NB:** Provision of study materials or patients.

**MA and FI:** Collection of data, analysis and interpretation.

**MA, NB and OS:** Manuscript writing and final approval of manuscript.
